# Combining aggregate and individual-level data to estimate individual-level associations between air pollution and COVID-19 mortality in the United States

**DOI:** 10.1371/journal.pgph.0002178

**Published:** 2023-08-02

**Authors:** Sophie M. Woodward, Daniel Mork, Xiao Wu, Zhewen Hou, Danielle Braun, Francesca Dominici

**Affiliations:** 1 Department of Biostatistics, Harvard T.H. Chan School of Public Health, Boston, Massachusetts, United States of America; 2 Department of Biostatistics, Columbia University, New York, New York, United States of America; 3 Department of Statistics, Columbia University, New York, New York, United States of America; 4 Department of Data Science, Dana-Farber Cancer Institute, Boston, Massachusetts, United States of America; Ashoka University, INDIA

## Abstract

Imposing stricter regulations for PM_2.5_ has the potential to mitigate damaging health and climate change effects. Recent evidence establishing a link between exposure to air pollution and COVID-19 outcomes is one of many arguments for the need to reduce the National Ambient Air Quality Standards (NAAQS) for PM_2.5_. However, many studies reporting a relationship between COVID-19 outcomes and PM_2.5_ have been criticized because they are based on ecological regression analyses, where area-level counts of COVID-19 outcomes are regressed on area-level exposure to air pollution and other covariates. It is well known that regression models solely based on area-level data are subject to ecological bias, i.e., they may provide a biased estimate of the association at the individual-level, due to within-area variability of the data. In this paper, we augment county-level COVID-19 mortality data with a nationally representative sample of individual-level covariate information from the American Community Survey along with high-resolution estimates of PM_2.5_ concentrations obtained from a validated model and aggregated to the census tract for the contiguous United States. We apply a Bayesian hierarchical modeling approach to combine county-, census tract-, and individual-level data to ultimately draw inference about individual-level associations between long-term exposure to PM_2.5_ and mortality for COVID-19. By analyzing data prior to the Emergency Use Authorization for the COVID-19 vaccines we found that an increase of 1 *μg*/*m*^3^ in long-term PM_2.5_ exposure, averaged over the 17-year period 2000-2016, is associated with a 3.3% (95% credible interval, 2.8 to 3.8%) increase in an individual’s odds of COVID-19 mortality. Code to reproduce our study is publicly available at https://github.com/NSAPH/PM_COVID_ecoinference. The results confirm previous evidence of an association between long-term exposure to PM_2.5_ and COVID-19 mortality and strengthen the case for tighter regulations on harmful air pollution and greenhouse gas emissions.

## Introduction

It is well known that greenhouse gases (GHG) and fine particulate matter (PM_2.5_) share the same emission sources (e.g. coal-fired power plants and diesel-fueled vehicles) [1, 2]. Therefore, implementing air quality control strategies in the United States will have the additional benefit of GHG emission reduction. On January 6, 2023, the EPA announced a proposal to strengthen the NAAQS for PM_2.5_ to better protect communities, with a focus on vulnerable populations who may be disproportionately impacted by the effects of air pollution [3, 4]. The EPA’s proposal specifically aims to lower the primary (health-based) annual PM_2.5_ standard from a level of 12 *μg*/*m*^3^ to a level between 9 and 10 *μg*/*m*^3^, taking into account the most up-to-date science. A growing body of evidence has established the association between exposure to PM_2.5_ and adverse health outcomes including heart disease, stroke, chronic obstructive pulmonary disease, lung cancer, and respiratory infections, among others [1]. The impacts of PM_2.5_ also extend to more recent health concerns including adverse COVID-19 outcomes based on studies both within the US [5] and worldwide [6].

The majority of the studies linking long-term PM_2.5_ exposure to COVID-19 health outcomes have relied on analyses of aggregated data [7], since publicly available data on COVID-19 outcomes are, for the most part, only available at aggregate-level (e.g., Johns Hopkins Coronavirus Resource Center or New York Times COVID-19 Tracker) [8, 9]. One of the first studies published on this topic analyzed county-level data for 3,082 US counties for the period March 1 to June 18, 2020, and found that an increase of 1 *μg*/*m*^3^ in long-term exposure (averaged during the years 2000–2016) to PM_2.5_ was significantly associated with an 11% (95% confidence interval, 6 to 17%) increase in the risk of COVID-19 death [5]. However, a major limitation of the aforementioned study is that it solely relied on county-level data [10, 11], so its conclusions cannot be interpreted as individual-level associations. Confusion between ecological associations and individual-level associations may present an ecological fallacy [12], which leads to associations detected in ecological regressions that do not exist or are in the opposite direction of true associations at the individual-level (i.e., ecological bias) [13–15]. To our knowledge, no study to date has performed an analysis of nationally representative data in the United States investigating the individual association between exposure to PM_2.5_ and COVID-19 mortality.

To overcome the challenge of ecological bias, a few studies in the United States have acquired and analyzed individual-level data on COVID-19 patients within specific subgroups or geographical locations. Up to January 5, 2023, we found a few US cohort studies, in which researchers accessed the University of Cincinnati healthcare system, the US Department of Veterans Affairs national healthcare databases, and Kaiser Permanente Southern California electronic medical records (EMR), to investigate the individual-level association between air pollution and COVID-19 outcomes [16–20]. Specifically, Mendy et al. (2021) acquired data on 14, 783 COVID-19 patients diagnosed at the University of Cincinnati healthcare system and found a significant positive association between long-term PM_2.5_ exposure and hospitalization among COVID-19 patients with asthma or COPD, but an insignificant association among patients without asthma or COPD [16]. Bowe et al. (2021) accessed individual-level data from the US Department of Veterans Affairs national healthcare databases and built a national cohort of 169, 102 veterans who tested positive for COVID-19. The authors found that long-term exposure to higher levels of PM_2.5_ was significantly associated with an increased risk of COVID-19 hospitalization [17]. Chen et al. (2022), Jerrett et al. (2023), and Sidell et al. (2022), analyzed COVID-19 outcomes among 4.6 million members of the Kaiser Permanente Southern California and found that short- and long-term PM_2.5_ exposure was significantly associated with increased risk of COVID-19 disease incidence, severity, and mortality [18–20]. These are powerful studies, but they include highly selective study populations and rely on health records that are not publicly available. Three additional studies worldwide have estimated associations between exposure to PM_2.5_ and COVID-19 outcomes using finer spatial units [21–23], the first using data at the census tract-level in Colorado, US, and the second and third using data at the level of small areas designed to be of similar population size in England and Scotland, respectively. These three studies implemented Bayesian hierarchical models with spatially-structured random effects. Although these studies may mitigate ecological bias by considering small spatial units, they do not harmonize data at different levels of spatial aggregation and do not incorporate any individual-level data. In this paper, we present the first and only epidemiological study in the US that adjusts for ecological bias and provides a more rigorous estimate of the *individual-level association* between long-term exposure to PM_2.5_ and COVID-19 mortality.

## Materials and methods

We begin by briefly summarizing our contributions. First, we harmonize and link nationally-representative US data from several publicly available sources at different levels of spatial aggregation. More specifically we link and harmonize data at the county-, gridded-, and individual-level to create a dataset containing the following: 1) COVID-19 deaths (county-level) which is our outcome; 2) exposure to air pollution (PM_2.5_) (census tract-level); 3) potential confounders including demographics and socioeconomic variables (individual-level); and 4) other county-level variables as potential confounders. Table 1 summarizes the data, the data sources, and their spatial resolution. Second, we implement a Bayesian hierarchical model to draw inference on individual-level associations between long-term PM_2.5_ exposure and COVID-19 mortality by combining information across individual-level, census tract-level, and county-level data [15]. Third, we update our previously constructed data repository for the period March 1 to June 18, 2020 [5], with data up to December 1, 2020, before the Emergency Use Authorization (EUA) for the Pfizer-BioNTech COVID-19 Vaccine, and implement both the proposed Bayesian hierarchical models and ecological regression models on the updated data. Fourth, we conduct extensive sensitivity analyses to examine the sensitivity of our results to different specifications of the statistical models. Finally, we make all data and code publicly available at https://doi.org/10.7910/DVN/3ZU0AS and https://github.com/NSAPH/PM_COVID_ecoinference, so other researchers can reproduce our analyses or apply a similar modeling framework to their data.

**Table 1:** Details of the data analyzed using our proposed Bayesian hierarchical model for combining sources at multiple spatial resolutions.

Type	Source	Description
*County-level outcome (3,082 counties)*		
COVID-19 deaths	Johns Hopkins University the Center for Systems Science and Engineering (JHU-CSSE) Coronavirus Resource Center	County-level COVID-19 death count from March 1 to December 1, 2020
*County-level covariates (10 covariates, 3,082 counties)*		
Average nitrogen dioxide (NO_2_)	NASA Socioeconomic Data and Applications Center (SEDAC) [24]	Average NO_2_ obtained by aggregating 1km×1km grid NO_2_ estimates for the contiguous United States across the period 2000–2016
Average ozone (O_3_)	NASA SEDAC [25]	Average O_3_ obtained by aggregating 1km×1km grid O_3_ estimates, for the contiguous United States across the period 2000–2016
Population density	US Census and American Community Survey (ACS)	Estimates in 2019
Hospital beds	Homeland Infrastructure Foundation Level Data (HIFLD)	Number of hospital beds in 2019
Average summer temperature, average winter temperature, average summer relative humidity, average winter relative humidity	Gridmet via Google Earth Engine	Weather variables obtained by aggregating 4km × 4km grid values across the period 2000–2016
Average % obese, % smokers	Robert Wood Johnson Foundation County Health Rankings	Behavioral risk factor variables for 2020
*Census tract-level exposure (72,538 census tracts)*		
Fine particulate matter (PM_2.5_)	Atmospheric Composition Analysis Group	Census tract average PM_2.5_ obtained by aggregating 0.01° × 0.01° grid estimates of PM_2.5_, for the contiguous United States across the period 2000–2016
*Individual-level covariates (8 covariates, 15,947,624 individuals, 7,613,443 households)*		
Poverty, high school graduation, owner-occupied, age, sex, race, household income, house value	Public Use Microdata Sample (PUMS) from ACS	A nationally representative sample of ACS responses, combined from years 2015–2019, which includes individual-level and household-level variables.

### Exposure data

Our primary exposure of interest is fine particulate matter (PM_2.5_). Consistent with Wu et al. [5], we use PM_2.5_ exposure data from an extensively cross-validated exposure prediction model [26], which predicts monthly PM_2.5_ exposure levels by fusing PM_2.5_ measures from three different sources: ground-based monitors, GEOS-Chem chemical transport models (CTM), and satellite observations at 0.01° × 0.01° grid resolution across the entire contiguous United States. We obtain long-term temporally averaged PM_2.5_ (2000–2016) by averaging the monthly concentrations. We used zonal statistics to aggregate the 0.01° × 0.01° gridded estimates of PM_2.5_ concentration to the census tract-level. To adjust for ecological bias, we additionally estimate the population-weighted variance of PM_2.5_ within each county. To do so, we first obtain the population sizes of all census tracts (which are much finer than counties), and then calculate the weighted variance of PM_2.5_ concentration in each county, incorporating census tract population sizes as the weights.

### Mortality data

We obtain and aggregate COVID-19 daily death reports for each county in the United States from the Center for Systems Science and Engineering at Johns Hopkins University for the period March 1 to December 1, 2020 (prior to the EUA for the Pfizer-BioNTech COVID-19 Vaccine) [8]. It is important to note that mortality across states may have been affected by state-level policies and reporting [27].

### County-level confounders

We treat long-term exposure to NO_2_ and O_3_ as county-level potential confounders. We use well-validated prediction models to estimate exposure to NO_2_ and O_3_ at the daily level from 2000 to 2016 at 1 km^2^ grids across the entire contiguous United States [24, 25]. We obtain long-term temporally averaged NO_2_ and O_3_ concentrations (from 2000 to 2016) by aggregating these concentrations to counties and averaging daily concentrations across these years. In this paper, we focus on PM_2.5_ exposure but similar methods could be used to extend the model to account for the within-county variability of NO_2_ and O_3_ exposure as well.

In addition to long-term exposure to NO_2_ and O_3_, we acquire another eight county-level variables to account for possible confounding bias at the county-level. Specifically, we consider two behavioral risk factor variables from the Robert Wood Johnson Foundation’s 2020 County Health Rankings: the proportion of residents who are obese and the proportion of residents who are current smokers; and four meteorological variables from Gridmet via Google Earth Engine: average daily temperature and relative humidity for summer (June-September) and winter (December-February) for each county [28, 29]. Finally, we obtain county-level population density estimates from the 2019 ACS, and the county-level number of hospital beds in 2019 from Homeland Infrastructure Foundation-Level Data.

### Public Use Microdata Sample (PUMS) data

We accessed a nationally representative data set with individual-level covariate data from the 5-Year Public Use Microdata Area (PUMA)-level PUMS files for the years 2015–2019, from the US Census Bureau’s ACS. The PUMS files are a set of records from individual people or housing units. PUMS files covering a five-year period contain data on approximately 5% of the United States population. The most detailed unit of geography in the PUMS files is the Public Use Microdata Area, which we link to US counties. For more details see [30] and S1 File. In our study, we consider eight individual-level demographic, social, and economic covariates from US Census Bureau’s ACS. These include six categorical covariates: sex, age, race, graduation status, house ownership, and poverty status; and two continuous covariates: household income and house value. To account for differences in COVID-19 mortality risks across age, race, and socioeconomic groups, we define 96 strata characterized by the joint distribution of the six individual-level categorical variables. Each stratum is determined by a unique combination of the values of categorical covariates: male/female, age 0–39/40+, White/Black/other race, graduated/not graduated from high school, owns/does not own house, and in poverty/not in poverty.

### Statistical methods

#### Bayesian hierarchical model

We define a Bayesian hierarchical model to estimate the individual-level association between long-term exposure to PM_2.5_ and COVID-19 deaths. This model builds upon the work by Jackson et al. (2006, 2008) with the goal of making inferences about individual-level relationships by combining aggregate- and individual-level data [15, 31]. The coefficients of this Bayesian hierarchical model are **consistent** with an underlying individual-level logistic model regressing the (unobserved) binary indicator of the outcome, COVID-19 death, for each individual to other independent variables, only requiring area-level death counts as the dependent variables.

To illustrate the mathematical details of our hierarchical model, we first introduce the underlying individual-level logistic regression model. Let *j* = 1, …, *n*_*i*_ index the individuals in county *i* = 1, …, *N*. The binary indicator, *y*_*ij*_, of COVID-19 related death for an individual *j* in county *i* can be modeled as

$$\begin{array}{cc} y_{ij} & ∼\text{Bernoulli}(p_{ij}),\\ \text{logit}(p_{ij}) & =\mu_{s_{i}}+x_{i}^{T}\alpha +v_{ij}^{T}\beta +\sum_{k=1}^{K}I_{ijk}\gamma_{k}, \end{array}$$ (1)

where *p*_*ij*_ is the probability of COVID-19 death; $\mu_{s_{i}}$ is the state-level random intercept for the state corresponding to county *i*; ***x***_***i***_ is a vector of county-level covariates for county *i* with linear effect coefficients ***α***; ***v***_***ij***_ is a vector of individual-level continuous covariates (e.g., PM_2.5_, log household income, log house value); *I*_*ijk*_ is an indicator that is equal to 1 if the individual *j* in county *i* belongs to stratum *k*; and *γ*_*k*_ is the effect for stratum *k*. We include state-specific random intercepts to control for differences in state-level policies and reporting practices. As described in the PUMS data section, we define 96 strata characterized by the joint distribution of the six individual-level categorical variables sex, age, race, graduation status, house ownership, and poverty status. Note that PM_2.5_ exposure is estimated on a high-resolution spatial grid and aggregated to the census tract-level, but we considered it here as an individual-level continuous variable under the assumption that individuals within the same census tract have similar long-term PM_2.5_ exposure. Using estimated PM_2.5_ at the census tract-level enables us to incorporate information about its within-county variance in the model.

The individual-level outcome data on COVID-19 deaths *y*_*ij*_, individual-level continuous covariates ***v***_***ij***_, and individual-level strata indicators *I*_*ijk*_ are unobserved, which prevents us from fitting the logistic regression model described in Eq 1 directly. However, by harnessing our individual-level data to estimate the within-county distribution of ***v***_***ij***_, we are able to fit a hierarchical model to estimate the individual-level association between long-term PM_2.5_ exposure and COVID-19 mortality. Although for certain highly selective patient groups we might access individual-level death records via EMR, in general *y*_*ij*_ is not publicly available for most US counties. Instead, we observe county-level COVID-19 deaths, $y_{i}=\sum_{j=1}^{n_{i}}y_{ij}$, for the whole contiguous United States. To harness individual-level covariate information into a statistical model with an aggregated outcome, we propose the hierarchical model,

$$\begin{array}{cc} y_{i} & ∼\text{Binomial}(n_{i},p_{i})\\ p_{i} & =\sum_{k=1}^{K}{\hat{\phi }}_{ik}q_{ik}\\ q_{ik} & =\int \text{expit}\left(\mu_{s_{i}}+x_{i}^{T}\alpha +\gamma_{k}+v^{T}\beta \right)g_{i}\left(v\right)dv, \end{array}$$

where *p*_*i*_ defines the marginal probability of COVID-19 death for county *i*, *q*_*ik*_ is the probability of COVID-19 death for an individual in county *i* who belongs to stratum *k*, ${\hat{\phi }}_{ik}$ is the proportion of individuals in county *i* belonging to stratum *k* estimated based on the PUMS data sample, and *g*_*i*_ denotes the joint density of the individual-level continuous covariates (PM_2.5_, log household income, log house value) within county *i*, which we assume is multivariate normal with estimated mean vector ***v***_***i***_ and estimated covariance matrix ${\hat{\Sigma }}_{i}$. Within each county, we assume that PM_2.5_ is uncorrelated with household income and house value, hence four entries of ${\hat{\Sigma }}_{i}$ equal 0. Table 2 describes the notation in detail.

**Table 2:** Descriptions of the notation for parameters and data used in the Bayesian hierarchical model.

Notation	Description
*Parameters*	
*p*_*i*_	Probability of COVID-19 death in county *i*
*q*_*ik*_	Probability of COVID-19 death for strata *k* in county *i*
$\mu_{s_{i}}$	State-specific random intercept for state *s* that contains county *i*
***α***	Vector of log odds for county-level data
*γ*_*k*_	Log odds for strata *k*
***β***	Vectors of log odds for individual-level covariates
*Data (observed)*	
*y*_*i*_	COVID-19 deaths in county *i*
*n*_*i*_	Population of county *i*
${\hat{\phi }}_{ik}$	Estimated proportion of individuals in strata *k* in county *i*
$x_{i}^{T}$	Vector of county-level covariates
$v_{i}^{T}$	Vector of county averages of individual-level covariates
${\hat{\Sigma }}_{i}$	Estimated covariance matrix of county-level covariates *i*

Under the assumption that individual-level continuous covariates are normally distributed within each county, *q*_*ik*_ can be approximated as

$$\begin{array}{c} q_{ik}\approx \text{expit}(\frac{\mu_{s_{i}}+x_{i}^{T}\alpha +\gamma_{k}+v_{i}^{T}\beta }{\sqrt{1+c^{2}\beta^{T}{\hat{\Sigma }}_{i}\beta }}), \end{array}$$ (2)

where $c=16\sqrt{3}/15\pi$, based on the probit approximation of the logit link function [15]. We log-transformed house value and household income to satisfy the assumption of normality and visually checked this assumption using histograms of log house value, log household income, and PM_2.5_ in each county. The log-likelihood of the proposed hierarchical model is

$$\begin{array}{c} \ell (\mu_{s_{i}},\alpha ,\gamma_{k},\beta ;y_{i},n_{i},{\hat{\phi }}_{ik},x_{i},v_{i},{\hat{\Sigma }}_{i})=\sum_{i=1}^{N}(y_{i}\;\text{log}\left(p_{i}\right)+\left(n_{i}-y_{i}\right)\;\text{log}\left(1-p_{i}\right)). \end{array}$$ (3)

An important feature of this model is that by maximizing the likelihood function described above we can make inferences on parameters $\left\{\mu_{s_{i}}\alpha ,\gamma_{k},\beta \right\}$ in the individual-level logistic regression model. In other words, with this hierarchical model, we can infer individual-level association, even though only county-level outcomes (*y*_*i*_) on COVID-19 deaths are available.

#### Bayesian model specification and computation

We implement a Bayesian hierarchical model via R statistical software (version 3.5.1) using package rStan [32, 33]. For each of the regression coefficient parameters {***α***, ***β***}, except for female and age ≥ 40, we choose Gaussian priors to reflect a 95% prior belief that each odds ratio is between 1/5 and 5. To reduce bias related to weak identifiability [34], we choose Gaussian priors for the female coefficient and age ≥ 40 coefficient based on Provisional COVID-19 Deaths by Sex and Age from the National Center for Health Statistics [35]. For the state-specific intercept $\mu_{s_{i}}$, we assign a hierarchical prior allowing for a random state-specific baseline risk of COVID-19 death. See S2 File for complete details on prior selection.

We incorporate *K* = 96 strata defined by six individual-level categorical covariates in our statistical model. To avoid identifiability issues and reduce the number of parameters, we assume additive effects of individual-level categorical covariates. Under this additivity assumption, each stratum *k* can be represented by a unique combination of *I*_*k*1_, …, *I*_*k*7_, where *I*_*kh*_ is an indicator for whether an individual in strata *k* belongs to subgroup *h* ∈ {1, …, 7}. Each subgroup is defined by the following individual-level categorical covariates: poverty, high school graduation, house ownership, age ≥ 40, female, Black, and other race respectively (note that the race variable is defined by two indicators since it contains three levels (White/Black/other race)). This allows us to represent each *γ*_*k*_ as a unique combination of parameters *ω*_*h*_ where $\gamma_{k}=\sum_{h=1}^{7}\omega_{h}I_{kh}$. As an example, the stratum corresponding to people in poverty, who had graduated from high school, did not own their house, are younger than 40, male, and identify as “other race” (not Black or White) would be attributed an individual-level effect of *γ*_*k*_ = *ω*_1_ + *ω*_2_ + *ω*_7_.

We used rStan to fit the model. We ran four chains, a total of 4,000 iterations each, with the first 2,000 iterations discarded as burn-in. All chains showed evidence of convergence after burn-in ($\hat{R}\leq 1.02$ for each parameter of concern) and produced similar coefficient estimates, which were combined to produce the final results [36].

#### Additional analyses

Along with the main analysis, we conduct three additional analyses to make comparisons that allow us to quantify ecological bias. First, we fit an ecological regression analysis to our dataset to compare alongside our main analysis. Second, we apply our Bayesian hierarchical model to a subset of the data extending only until June 18, 2020, to match the data used in the previous published ecological regression [5]. As Wu et. al. (2020) utilized a different modeling approach for the reduced dataset, we also conduct an ecological regression analysis to the dataset extending through June 18, 2020 to quantify ecological bias.

#### Sensitivity analyses

To evaluate the robustness of the estimated odds ratios for PM_2.5_ to various modeling specifications, we conduct a series of sensitivity analyses. First, we account for four categories in age (0–17, 18–39, 40–64, 65+) instead of two (0–39, 40+) to more accurately adjust for variations in COVID-19 mortality by age. Second, we use county-level deaths and the PUMS data sample to estimate fixed values for the strata specific risk coefficients, *γ*_*k*_. Using fixed values as opposed to estimating the strata-specific coefficients allows us to consider potential bias in the categorical offsets related to weak identifiability. Third, we include PM_2.5_ as a county-level covariate in the Bayesian hierarchical model, similar to NO_2_ and O_3_, to determine the robustness of the estimated ORs for PM_2.5_ if the within-county variability of the exposure was not taken into account. We run all combinations of the proposed sensitivity analyses and compare to our main analysis.

## Results

**Fig 1:**
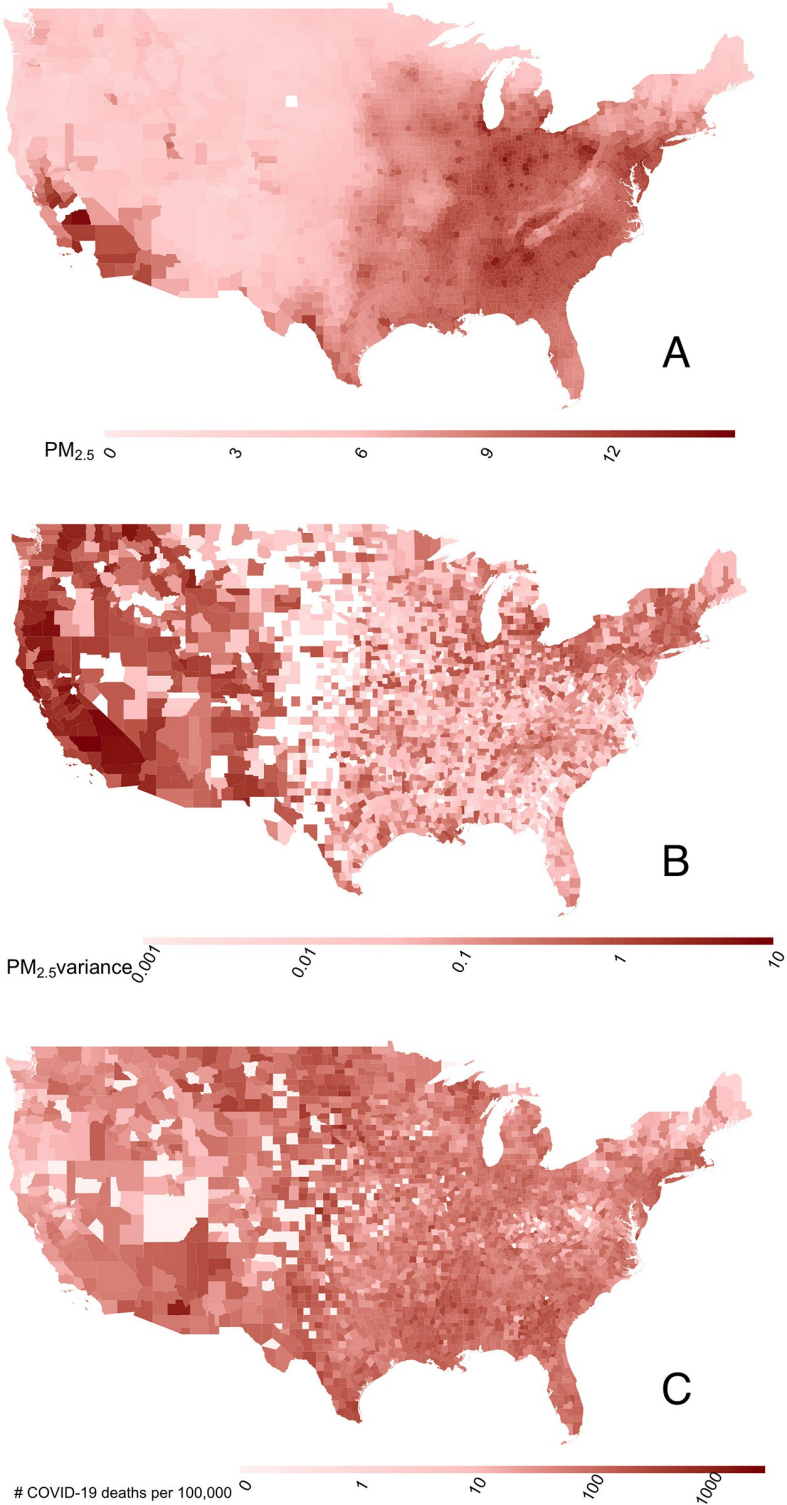
Maps showing (a) county-level 17-year average of PM_2.5_ concentrations (2000–2016) in *μg*/*m*^3^, (b) estimated population-weighted variance of the census tract-level PM_2.5_ concentrations (2000–2016) within each county, and (c) county-level COVID-19 death counts per 100,000 individuals in the United States up to December 1, 2020. Estimated variances of PM_2.5_ are equal to zero in 6% of counties because they only contain one census tract. Base geography layers retrieved from U.S. Census TIGER/Line shapefiles: https://www.census.gov/geographies/mapping-files/time-series/geo/tiger-line-file.html.

Fig 1 shows: 1) county-level 17-year average PM_2.5_ concentrations (2000–2016) in the United States in *μg*/*m*^3^; 2) estimated population-weighted variance of the census tract-level PM_2.5_ concentrations (2000–2016) within each county; and 3) county-level number of COVID-19 deaths per 100,000 individuals in the United States for the period March 1 to December 1, 2020. These maps show higher PM_2.5_ levels in the southwest and southeast; higher PM_2.5_ variances in the west; and higher COVID-19 death rates in the southwest, southeast and mid-west. In counties with higher exposure variances, county-level average exposure to PM_2.5_ is less representative of the census tract aggregated exposure. Indeed, it is well known that as the ratios of within-county to between-county variability of exposure and confounders increase, ecological bias increases [15, 31].

Table 3 summarizes the characteristics of the study cohort. In Table 4, we report the estimated regression coefficient for each of the covariates included in our main analysis. In our main analysis, we consider a study period of March 1 to December 1, 2020, and account for ecological and confounding bias by incorporating individual-level covariate data from PUMS and census tract-level exposure data for PM_2.5_ in the proposed Bayesian hierarchical model. We found that an increase of 1 *μg*/*m*^3^ in long-term average PM_2.5_ is associated with an increase in an individual’s odds of COVID-19 death by 3.3% (95% credible interval (CI): 2.8% to 3.8%), after adjusting for both ecological and confounding bias. Importantly, we also found that county-level nitrogen dioxide and ozone (NO_2_ and O_3_), population density, rate of hospital beds, average summer and winter temperature, average summer and winter relative humidity, obesity, smoking status, and individual-level poverty status, high school graduation status, house ownership, age, sex, race, household income, and house value are significantly associated with COVID-19 mortality rate.

**Table 3:** Characteristics of the study cohort. For census tract-level data, the mean and standard deviation across all US census tracts is calculated weighted by the populations in the census tracts. For county-level data, the mean and standard deviation across all US counties is calculated. For individual-level categorical data, the percent of individuals in each category is calculated. For individual-level continuous data, the mean and standard deviation across all individuals in the Public Use Microdata Sample is calculated, using accompanying sampling weights provided by American Community Survey (ACS). Other race denotes races other than White and Black.

Covariate	Mean (SD) or %
*Census tract-level data*	
Average PM_2.5_ (*μg*/*m*^3^)	7.9 (2.2)
*County-level data*	
COVID-19 death rate (per 100,000)	82.8 (75.6)
Average NO_2_	12.8 (5.1)
Average O_3_	40.1 (2.6)
Rate of hospital beds (per 100,000)	303.9 (428.2)
Population density	234.1 (1519.6)
Average summer temperature (℃)	30.0 (3.2)
Average winter temperature (℃)	7.2 (6.6)
Average summer relative humidity (%)	89.0 (9.6)
Average winter relative humidity (%)	87.5 (4.8)
% Obese	33.0 (5.4)
% Smoke	17.4 (3.5)
*Individual-level data*	
Poverty	13.1
High school graduation	70.7
Owner-occupied housing	63.7
Age ≥ 40	47.6
Female	50.8
Black	12.7
Other race	14.8
Household income ($1000)	88.4 (97.0)
House value ($1000)	307.1 (408.5)

**Table 4:** COVID-19 mortality odds ratios and 95% credible intervals (CI) for all covariates, accounting for ecological and individual-level confounding bias using our proposed Bayesian hierarchical model. Odds ratios for individual-level categorical variables can be interpreted relative to the following baseline levels: not in poverty, not graduated from high school, does not own house, age 0−39, male, and White race. For population density, Q denotes quintile with coefficients relative to Q1. Other race denotes races other than White and Black.

Covariate	Odds Ratio	95% CI
*Census tract-level data*		
PM_2.5_ (*μg*/*m*^3^)	1.033	(1.028, 1.038)
*County-level data*		
NO_2_	1.011	(1.009, 1.012)
O_3_	0.982	(0.979, 0.985)
Population density (Q2)	0.876	(0.842, 0.911)
Population density (Q3)	0.820	(0.790, 0.852)
Population density (Q4)	0.730	(0.703, 0.758)
Population density (Q5)	0.767	(0.738, 0.798)
Rate of hospital beds	1.076	(1.067, 1.084)
Average summer temperature (℃)	1.175	(1.156, 1.195)
Average winter temperature (℃)	1.064	(1.037, 1.092)
Average summer relative humidity (%)	0.966	(0.951, 0.982)
Average winter relative humidity (%)	0.983	(0.974, 0.992)
% Obese	1.048	(1.039, 1.057)
% Smoke	0.935	(0.923, 0.946)
*Individual-level data*		
Poverty	3.580	(3.195, 4.012)
High school graduation	0.103	(0.095, 0.112)
Owner-occupied	3.035	(2.770, 3.325)
Age ≥ 40	56.204	(54.28, 58.197)
Female	0.649	(0.612, 0.689)
Black	1.167	(1.111, 1.225)
Other race	1.641	(1.543, 1.744)
log(household income)	0.728	(0.671, 0.791)
log(house value)	1.262	(1.219, 1.306)

In Table 5, we report the estimated OR and 95% CI for PM_2.5_ under the following three additional analyses described in the Methods section: 1) ecological regression analysis for the whole study period (March 1 to December 1, 2020) not accounting for ecological bias (i.e., using county-level data only); 2) analysis accounting for ecological bias, but for a shorter time period (March 1 to June 18, 2020, to align with the previous analysis [5]); 3) ecological regression analysis not accounting for ecological bias for the time period of March 1 to June 18, 2020. We compare the results from these three additional analyses with the results from the main analysis (the estimated coefficients for the main analysis are shown in Table 4). There are three important findings. First, the estimated OR for PM_2.5_ for the shorter period (up to June 18, 2020) is larger than the estimated OR for the longer study period (up to December 1, 2020). Second, adjusting for ecological bias by incorporating individual-level confounders and census tract-level PM_2.5_ reduces the estimated OR. However, the smaller OR reported in the main analysis is mostly due to updating the data up to December 2020 and less due to the adjustment for ecological bias. Overall, all four estimated ORs show statistically significant positive county-level/individual-level associations between long-term exposure to PM_2.5_ and COVID-19 mortality.

**Table 5:** Summary of COVID-19 mortality odds ratios and 95% credible intervals (CI) for a 1 *μg*/*m*^3^ increase in PM_2.5_ from additional analyses. The first row represents our main analysis with individual-level confounders and census tract-level PM_2.5_ accounting for ecological and confounding bias (up to December 1, 2020). The second, third, and fourth rows represent the three additional analyses respectively: ecological regression with solely county-level data (up to December 1, 2020); Bayesian hierarchical regression with individual-level confounders and census tract-level PM_2.5_; and ecological regression with solely county-level data (up to June 18, 2020).

Model	Data End Date	Odds Ratio	95% CI
Hierarchical	12/1/2020	1.033	(1.028, 1.038)
Ecological	12/1/2020	1.051	(1.046, 1.055)
Hierarchical	6/18/2020	1.099	(1.089, 1.110)
Ecological	6/18/2020	1.105	(1.096, 1.115)

We conduct several sensitivity analyses, where we compare the results from our main analysis with results under seven additional model specifications and implementations described in the Methods section. We find results from our sensitivity analyses are overall consistent with results from the main analysis. In particular, we find statistically significant positive associations between long-term exposure to PM_2.5_ and the odds of COVID 19 death, regardless of the model specification (see Table 6 for the results).

**Table 6:** Summary of COVID-19 mortality odds ratios (OR) and 95% credible intervals (CI) for a 1 *μg*/*m*^3^ increase in PM_2.5_ from sensitivity analyses based on 1) changing the number of age groups used for stratification, 2) using fixed versus estimated offsets for strata-specific odds ratios, and 3) estimating PM_2.5_ aggregated to the county-level or census tract-level. The first row represents our main analysis.

# Age groups	Strata Offsets	PM_2.5_ Resolution	OR	95% CI
2	Estimated	Census tract	1.033	(1.028, 1.038)
2	Fixed	Census tract	1.044	(1.039, 1.049)
4	Estimated	Census tract	1.037	(1.032, 1.043)
4	Fixed	Census tract	1.041	(1.036, 1.046)
2	Estimated	County	1.033	(1.028, 1.038)
2	Fixed	County	1.044	(1.039, 1.049)
4	Estimated	County	1.037	(1.032, 1.042)
4	Fixed	County	1.041	(1.035, 1.046)

## Discussion

Our work presents the first study using nationally representative and publicly available data in the United States to investigate relationships between air pollution exposure and COVID-19 mortality and make inference at the individual-level. Our statistical model adjusts for a range of socioeconomic, demographic, meteorological, behavioral, and health-related confounders. Compared to an ecological regression model, our Bayesian hierarchical model adjusts for ecological bias by incorporating individual-level data and produces estimates that continue to indicate strong evidence of a harmful relationship between increased PM_2.5_ exposure and odds of COVID-19 mortality. Our results are robust under a variety of assumptions considered in sensitivity analyses.

We found that when we analyze the data for the longer study period (March 1 to December 1, 2020) the estimated OR is smaller than when we analyze the data for a shorter period (March 1 to June 18, 2020). The smaller association in the longer period compared to the shorter period may be due to many reasons, including non-pharmaceutical interventions, changes in mobility and lifestyle, and other factors that we did not control for in our analysis [20]. It should also be noted that in addition to having higher pollution levels, larger cities tend to have stronger international connections, which facilitated spread of the virus during the early stages of the pandemic [6]. Adjusting for population density alone may not have fully accounted for this phenomenon.

Although the exact mechanism explaining a causal relationship between higher levels of air pollution exposure and increased COVID-19 mortality remains unclear, previous work studied the potential biological links between air pollution and COVID-19 mortality. First, COVID-19 spread occurs via airborne particles and droplets, and PM_2.5_ can create a suitable environment, which increases the distance of COVID-19 transmission [37–41]. Second, PM_2.5_ can induce cellular inflammation, and exposure to such particles can exacerbate the susceptibility and severity of symptoms among COVID-19 patients [37, 42–45]. Third, it is also found that exposure to air pollution may suppress early immune responses to the infection, which leads to later exacerbation in inflammation and worse prognosis [46–51]. For example, long-term exposure to PM_2.5_ and NO_2_ leads to the overexpression of the angiotensin converting enzyme 2 (ACE-2), and this overexpression relates with the severity in COVID-19 patients [37, 52]. We maintain that our results do not imply a causal effect of air pollution, but existing work suggests that this pathway could exist.

Several studies have examined the association between COVID-19 outcomes and air pollution, including ecological regressions in different countries [5, 53–60], cohort studies including individual-level data for specific populations around the world [17, 61–71], and Bayesian hierarchical models that attempt to overcome the clustered nature of the data [21, 22]. In addition, literature reviews have been published on the topic [6, 72–77] as well as a meta-analysis [78]. Most of these studies reported evidence of a statistically significant association between exposure to air pollution and COVID-19 incidence and mortality risk. However, to our knowledge, none of these studies leverage a large and nationally representative sample of individual-level confounders and census tract-level PM_2.5_ to estimate the association between exposure to air pollution and COVID-19 mortality in the contiguous United States accounting for both confounding and ecological bias.

In this nationwide study, we combine county-level, census tract-level and individual-level information from a wide range of data sources to infer individual-level associations between long-term exposure to PM_2.5_ and the odds of COVID-19 death adjusting for potential confounding and ecological bias. Compared with an ecological regression analysis which only relies on county-level data of these variables, our analysis quantifies and adjusts for ecological bias by incorporating a nationally representative sample of demographic, social, and economic variables from the 2015–2019 American Community Survey (ACS) Public Use Microdata Sample (PUMS). Due to data privacy constraints, it is increasingly challenging to collect a large amount of nationally representative individual-level data relevant to COVID-19 outcomes and make it publicly available in a timely manner [79]. Our proposed Bayesian method overcomes these constraints in a novel way. It allows us to obtain unbiased estimates of individual-level associations when the COVID-19 outcome data is only available at the area-level by incorporating individual-level and finer spatial resolution data to account for within-area variations of both exposures and potential confounders. The approach implemented in this paper and the associated software can be applied to infer individual-level associations in any analysis that has access to individual-level confounders but only aggregate-level health data.

We have chosen to restrict our study period from March 1 to December 1, 2020. Extending our study to more recent data—and therefore post-vaccination—requires careful consideration due to a host of additional confounding factors. First, it is well documented that vaccines are highly effective at preventing hospitalizations and death [80–83]. However, controlling for vaccination rates is challenging because of the complex interaction between time and individual vaccination status as different groups became eligible to receive the COVID-19 vaccine at different times. Second, urban population centers tended to have higher vaccination rates compared to rural settings [84–86], which may serve to lower COVID-19 mortality, but urban areas are also associated with increased levels of air pollution [87], which would have the opposite relationship with COVID-19 mortality if our results hold. Hence, without properly controlling for individual vaccination status we risk biasing the results of the analysis using data from post-vaccination periods.

There are still many limitations in our study, which we hope can be resolved or improved in the future. First, within-county variances of PM_2.5_ are estimated using gridded-level (0.01° × 0.01°) estimates, aggregated to the census tract-level and weighting by census tract population. While the estimated association of PM_2.5_ and odds of COVID-19 mortality applies to the individual, we only have estimates of PM_2.5_ at the census tract-level and not at the individual-level. However, while this is a limitation, we argue that the assumption that all individuals within the same census tract have the same long-term average exposure to PM_2.5_ is reasonable. Indeed, Currie et al. (2020) report that almost all variation in individual-level PM_2.5_ exposure can be explained by census tract characteristics [88]. Second, we make the strong assumption that the joint distribution of PM_2.5_ exposure, log household income, and log house value is multivariate normal within each county. Moreover, estimating the correlation of PM_2.5_ exposure with log household income and log house value is difficult, since individual-level PM_2.5_ exposures are not available, forcing us to make the unlikely assumption that PM_2.5_ exposure is uncorrelated with the other two variables within each county. Third, there exists some temporal misalignment in the exposure and confounders. However, considering that this is a cross-sectional analysis and exposure is averaged over 17 years, we expect that our results would be robust to the inclusion of more recent data. Fourth, although we were able to infer individual-level associations accounting for several individual-level and county-level confounders, results could still be biased due to unmeasured confounding. Our study relies only on publicly available data, which contains limited information on individual-level risk factors. However, we note that a recent study relating air pollution exposure with all-cause, respiratory, and circulatory hospital admissions found results to be robust to the exclusion of numerous individual-level risk factors including smoking, body mass index, and pre-existing health conditions [89]. Additionally, a COVID-19 cohort study relating PM_2.5_ exposure and disease-related hospitalization risk did not find sensitivity to the inclusion of individual-level risk factors, including body mass index, cardiovascular disease, chronic obstructive pulmonary disease, diabetes, and other clinical factors [17]. Fifth, although we conduct sensitivity analyses, it remains possible that results are biased due to model misspecification. Sixth, gridded exposure estimates of PM_2.5_ concentrations are obtained from an exposure prediction model, and in this analysis, we ignore its associated statistical uncertainty. Finally, we were primarily interested in PM_2.5_ in this analysis, but our model could be extended to include within-area variability of NO_2_, O_3_, and other pertinent exposures in future work.

Considering how modifiable environmental factors alter the severity and mortality of COVID-19, among other heath outcomes, is key to helping guide public policies to reduce the negative impact of an epidemiological outbreak. This work provides updated evidence regarding the association between long-term exposure to PM_2.5_ and an individual’s odds of COVID-19 mortality in the United States. Our results are derived from population-level COVID-19 outcomes, individual-level confounder data from a large national sample of 15,947,624 individuals and 7,613,443 households, and high-resolution air pollution exposures predicted from well-validated models. In line with the findings from previous studies, our analysis reveals a statistically significant, albeit small, positive correlation between long-term exposure to PM_2.5_ and individual-level COVID-19 mortality. This discovery holds immense significance for public health, considering the cumulative impact of air pollution exposure on a large population. Combined with other work indicating the harmful impacts of PM_2.5_ exposure on health, we refocus our attention to the NAAQS for PM_2.5_ and related environmental policies. In particular, our results suggest that prior to the EUA for the Pfizer-BioNTech COVID-19 Vaccine, lowering the long-term annual average NAAQS for PM_2.5_ from 12*μg*/*m*^3^ to follow the World Health Organization recommendation of 5*μg*/*m*^3^ [90] is associated with a reduction in an individual’s odds of COVID-19 mortality by 20.0%. Since GHG and PM_2.5_ share the same emissions sources, implementing stricter regulations for PM_2.5_ will not only lead to enormous public health benefits but also to reductions of GHG, thereby mitigating additional damaging effects and enormous costs related to climate change.

## Supporting information

S1 File Public use microdata sample. (PDF)

S2 File Prior selection. (PDF)

## References

[1] US Environmental Protection Agency. Integrated Science Assessment (ISA) for Particulate Matter (Final Report). EPA. 2019;600/R-19/188.

[2] US Environmental Protection Agency. Inventory of U.S. Greenhouse Gas Emissions and Sinks: 1990-2020. EPA. 2022;430/R-22/003.

[3] US Environmental Protection Agency. Reviewing National Ambient Air Quality Standards (NAAQS): Scientific and Technical Information. EPA. 2021; EPA–HQ–OAR–2015–0072.

[4] US Environmental Protection Agency. EPA Proposes to Strengthen Air Quality Standards to Protect the Public from Harmful Effects of Soot. EPA. 2023.

[5] Wu X, Nethery RC, Sabath M, Braun D, Dominici F. Air pollution and COVID-19 mortality in the United States: Strengths and limitations of an ecological regression analysis. Science advances. 2020;6(45):eabd4049. doi: 10.1126/sciadv.abd4049 33148655 PMC7673673

[6] Bhaskar A, Chandra J, Hashemi H, Butler K, Bennett L, Cellini J, et al. A Literature Review of the Effects of Air Pollution on COVID-19 Health Outcomes Worldwide: Statistical Challenges and Data Visualization. Annual Review of Public Health. 2022;44. 36542771 10.1146/annurev-publhealth-071521-120424 PMC11567163

[7] Weaver AK, Head JR, Gould CF, Carlton EJ, Remais JV. Environmental factors influencing COVID-19 incidence and severity. Annual Review of Public Health. 2022;43:271–291. doi: 10.1146/annurev-publhealth-052120-101420 34982587 PMC10044492

[8] Dong E, Du H, Gardner L. An interactive web-based dashboard to track COVID-19 in real time. The Lancet infectious diseases. 2020;20(5):533–534. doi: 10.1016/S1473-3099(20)30120-1 32087114 PMC7159018

[9] Smith M, Yourish K, Almukhtar S, et al. An ongoing repository of data on coronavirus cases and deaths in the US New York Times;.

[10] Villeneuve PJ, Goldberg MS. Methodological considerations for epidemiological studies of air pollution and the SARS and COVID-19 coronavirus outbreaks. Environmental Health Perspectives. 2020;128(9):095001. doi: 10.1289/EHP7411 32902328 PMC7480171

[11] Barceló MA, Saez M. Methodological limitations in studies assessing the effects of environmental and socioeconomic variables on the spread of COVID-19: a systematic review. Environmental Sciences Europe. 2021;33(1):1–18. doi: 10.1186/s12302-021-00550-7 34522574 PMC8432444

[12] Sudweeks F, Herring S. Culture, technology, communication: Towards an intercultural global village. Suny Press; 2001.

[13] Richardson S, Stücker I, Hémon D. Comparison of relative risks obtained in ecological and individual studies: some methodological considerations. International journal of epidemiology. 1987;16(1):111–120. doi: 10.1093/ije/16.1.111 3570609

[14] Greenland S, Morgenstern H. Ecological bias, confounding, and effect modification. International journal of epidemiology. 1989;18(1):269–274. doi: 10.1093/ije/18.1.269 2656561

[15] Jackson C, Best N, Richardson S. Improving ecological inference using individual-level data. Statistics in medicine. 2006;25(12):2136–2159. doi: 10.1002/sim.2370 16217847

[16] Mendy A, Wu X, Keller JL, Fassler CS, Apewokin S, Mersha TB, et al. Air pollution and the pandemic: Long-term PM2. 5 exposure and disease severity in COVID-19 patients. Respirology. 2021;. doi: 10.1111/resp.14140 34459069 PMC8662216

[17] Bowe B, Xie Y, Gibson AK, Cai M, van Donkelaar A, Martin RV, et al. Ambient fine particulate matter air pollution and the risk of hospitalization among COVID-19 positive individuals: Cohort study. Environment International. 2021;154:106564. doi: 10.1016/j.envint.2021.106564 33964723 PMC8040542

[18] Chen Z, Sidell MA, Huang BZ, Chow T, Eckel SP, Martinez MP, et al. Ambient Air Pollutant Exposures and COVID-19 Severity and Mortality in a Cohort of COVID-19 Patients in Southern California. American Journal of Respiratory and Critical Care Medicine. 2022;(ja). 10.1164/rccm.202108-1909OC PMC12039156 35537137

[19] Jerrett M, Nau CL, Young DR, Butler RK, Batteate CM, Su J, et al. Air pollution and meteorology as risk factors for COVID-19 death in a cohort from Southern California. Environment International. 2023;171:107675. doi: 10.1016/j.envint.2022.107675 36565571 PMC9715495

[20] Sidell MA, Chen Z, Huang BZ, Chow T, Eckel SP, Martinez MP, et al. Ambient air pollution and COVID-19 incidence during four 2020–2021 case surges. Environmental research. 2022;208:112758. doi: 10.1016/j.envres.2022.112758 35063430 PMC8767981

[21] Berg K, R Present P, Richardson K. Long-term air pollution and other risk factors associated with COVID-19 at the census tract level in Colorado. Environmental Pollution. 2021;287:117584. doi: 10.1016/j.envpol.2021.117584 34153607 PMC8202820

[22] Konstantinoudis G, Padellini T, Bennett J, Davies B, Ezzati M, Blangiardo M. Long-term exposure to air-pollution and COVID-19 mortality in England: A hierarchical spatial analysis. Environment international. 2021;146:106316–106316. doi: 10.1016/j.envint.2020.106316 33395952 PMC7786642

[23] Lee D, Robertson C, McRae C, Baker J. Quantifying the impact of air pollution on Covid-19 hospitalisation and death rates in Scotland. Spatial and Spatio-temporal Epidemiology. 2022; p. 100523. doi: 10.1016/j.sste.2022.100523 35934329 PMC9176207

[24] Di Q, Amini H, Shi L, Kloog I, Silvern R, Kelly J, et al. Assessing NO2 concentration and model uncertainty with high spatiotemporal resolution across the contiguous United States using ensemble model averaging. Environmental science & technology. 2019;54(3):1372–1384. doi: 10.1021/acs.est.9b03358 PMC7065654 31851499

[25] Requia WJ, Di Q, Silvern R, Kelly JT, Koutrakis P, Mickley LJ, et al. An ensemble learning approach for estimating high spatiotemporal resolution of ground-level ozone in the contiguous United States. Environmental science & technology. 2020;54(18):11037–11047. doi: 10.1021/acs.est.0c01791 32808786 PMC7498146

[26] Van Donkelaar A, Martin RV, Li C, Burnett RT. Regional estimates of chemical composition of fine particulate matter using a combined geoscience-statistical method with information from satellites, models, and monitors. Environmental science & technology. 2019;53(5):2595–2611. doi: 10.1021/acs.est.8b06392 30698001

[27] Unwin HJT, Mishra S, Bradley VC, Gandy A, Mellan TA, Coupland H, et al. State-level tracking of COVID-19 in the United States. Nature Communications. 2020; doi: 10.1038/s41467-020-19652-6 33273462 PMC7712910

[28] Abatzoglou John T. Development of gridded surface meteorological data for ecological applications and modelling. International Journal of Climatology. 2013;. doi: 10.1002/joc.3413

[29] Gorelick N, Hancher M, Dixon M, Ilyushchenko S, Moore R. Google Earth Engine: Planetary-scale geospatial analysis for everyone. Remote Sensing of Environment. 2017;202. doi: 10.1016/j.rse.2017.06.031

[30] ACS. 2015-2019 5-YEAR Public Use Microdata Sample FILES ReadMe. US Census Bureau. 2021;.

[31] Jackson C, Best N, Richardson S. Hierarchical related regression for combining aggregate and individual data in studies of socio-economic disease risk factors. Journal of the Royal Statistical Society: Series A (Statistics in Society). 2008;171(1):159–178. doi: 10.1111/j.1467-985X.2007.00500.x

[32] R Core Team. R: A Language and Environment for Statistical Computing; 2020. Available from: https://www.R-project.org/.

[33] Gelman A, Lee D, Guo J. Stan: A probabilistic programming language for Bayesian inference and optimization. Journal of Educational and Behavioral Statistics. 2015;40(5):530–543. doi: 10.3102/1076998615606113

[34] Ogle K, Barber JJ. Ensuring identifiability in hierarchical mixed effects Bayesian models. Ecological Applications. 2020;30(7):e02159. doi: 10.1002/eap.2159 32365250

[35] National Center for Health Statistics. Provisional COVID-19 Deaths by Sex and Age; 2023. Available from: https://data.cdc.gov/d/9bhg-hcku.

[36] Gelman A, Rubin DB. Inference from iterative simulation using multiple sequences. Statistical science. 1992;7(4):457–472. doi: 10.1214/ss/1177011136

[37] Comunian S, Dongo D, Milani C, Palestini P. Air Pollution and COVID-19: The Role of Particulate Matter in the Spread and Increase of COVID-19 Morbidity and Mortality. International Journal of Environmental Research and Public Health. 2020;17(12). doi: 10.3390/ijerph17124487 32580440 PMC7345938

[38] Peng L, Zhao X, Tao Y, Mi S, Zhang Q. The effects of air pollution and meteorological factors on measles cases in Lanzhou, China. Environmental Science and Pollution Research. 2020;27(1). 32030582 10.1007/s11356-020-07903-4

[39] Ye Q, Fu JF, Mao JH, Shang SQ. Haze is a risk factor contributing to the rapid spread of respiratory syncytial virus in children. Environmental Science & Pollution Research. 2016;23(20):1–8. 27439752 10.1007/s11356-016-7228-6

[40] Nor NSM, Yip CW, Ibrahim N, Jaafar MH, Rashid ZZ, Mustafa N, et al. Particulate matter (PM 2.5) as a potential SARS-CoV-2 carrier. Scientific Reports. 2021;11(1):1–6. doi: 10.1038/s41598-021-81935-9 33510270 PMC7844283

[41] Pivato A, Amoruso I, Formenton G, Di Maria F, Bonato T, Vanin S, et al. Evaluating the presence of SARS-CoV-2 RNA in the particulate matters during the peak of COVID-19 in Padua, northern Italy. Science of the Total Environment. 2021;784:147129. doi: 10.1016/j.scitotenv.2021.147129 33894607 PMC8050405

[42] Frontera A, Martin C, Vlachos K, Sgubin G. Regional air pollution persistence links to COVID-19 infection zoning. Journal of Infection. 2020;81(2). doi: 10.1016/j.jinf.2020.03.045 32283151 PMC7151372

[43] Tsai DH, Riediker M, Berchet A, Paccaud F, Bochud M. Effects of short- and long-term exposures to particulate matter on inflammatory marker levels in the general population. Environmental Science and Pollution Research. 2019;26(4). 10.1007/s11356-019-05194-y 31079306

[44] Iii CAP, Bhatnagar A, Mccracken JP, Abplanalp W, Conklin DJ, O’Toole T. Exposure to Fine Particulate Air Pollution Is Associated With Endothelial Injury and Systemic InflammationNovelty and Significance. Circulation Research. 2016;. 10.1161/CIRCRESAHA.116.309279 PMC5215745 27780829

[45] Conticini E, Frediani B, Caro D. Can atmospheric pollution be considered a co-factor in extremely high level of SARS-CoV-2 lethality in Northern Italy? Environmental pollution. 2020;261:114465. doi: 10.1016/j.envpol.2020.114465 32268945 PMC7128509

[46] Ciencewicki J, Jaspers I. Air Pollution and Respiratory Viral Infection. Inhalation toxicology. 2007;19:1135–46. doi: 10.1080/08958370701665434 17987465

[47] Becker S, Soukup JM. Exposure to urban air particulates alters the macrophage-mediated inflammatory response to respiratory viral infection. Journal of Toxicology & Environmental Health Part A. 1999;57(7):445–457. 10494914 10.1080/009841099157539

[48] Upadhyay D, Panduri V, Ghio A, Kamp DW. Particulate matter induces alveolar epithelial cell DNA damage and apoptosis: role of free radicals and the mitochondria. American journal of respiratory cell and molecular biology. 2003;29(2):180–187. doi: 10.1165/rcmb.2002-0269OC 12600817

[49] Kelly F, Fussell J. Air pollution and airway disease. Clinical & Experimental Allergy. 2011;41(8):1059–1071. doi: 10.1111/j.1365-2222.2011.03776.x 21623970

[50] Mendy A, Wilkerson J, Salo PM, Weir CH, Feinstein L, Zeldin DC, et al. Synergistic association of house endotoxin exposure and ambient air pollution with asthma outcomes. American journal of respiratory and critical care medicine. 2019;200(6):712–720. doi: 10.1164/rccm.201809-1733OC 30965018 PMC6775869

[51] Mendy A, Wu X, Keller JL, Fassler CS, Apewokin S, Mersha TB, et al. Long-term exposure to fine particulate matter and hospitalization in COVID-19 patients. Respiratory medicine. 2021;178:106313. doi: 10.1016/j.rmed.2021.106313 33550152 PMC7835077

[52] Paital B, Agrawal PK. Air pollution by NO2 and PM2.5 explains COVID-19 infection severity by overexpression of angiotensin-converting enzyme 2 in respiratory cells: a review. Environmental Chemistry Letters. 2020; p. 1–18. 32982622 10.1007/s10311-020-01091-w PMC7499935

[53] Prinz AL, Richter DJ. Long-term exposure to fine particulate matter air pollution: an ecological study of its effect on COVID-19 cases and fatality in Germany. Environmental research. 2022;204:111948. doi: 10.1016/j.envres.2021.111948 34464613 PMC8400616

[54] Hendryx M, Luo J. COVID-19 prevalence and fatality rates in association with air pollution emission concentrations and emission sources. Environmental Pollution. 2020;265:115126. doi: 10.1016/j.envpol.2020.115126 32806422 PMC7320861

[55] Norouzi N, Asadi Z. Air pollution impact on the Covid-19 mortality in Iran considering the comorbidity (obesity, diabetes, and hypertension) correlations. Environmental Research. 2022;204:112020. doi: 10.1016/j.envres.2021.112020 34509488 PMC8426329

[56] Cole MA, Ozgen C, Strobl E. Air pollution exposure and Covid-19 in Dutch municipalities. Environmental and Resource Economics. 2020;76(4):581–610. doi: 10.1007/s10640-020-00491-4 32836849 PMC7399597

[57] Yao Y, Pan J, Wang W, Liu Z, Kan H, Qiu Y, et al. Association of particulate matter pollution and case fatality rate of COVID-19 in 49 Chinese cities. Science of the Total Environment. 2020;741:140396. doi: 10.1016/j.scitotenv.2020.140396 32592974 PMC7305499

[58] Liang D, Shi L, Zhao J, Liu P, Sarnat JA, Gao S, et al. Urban air pollution may enhance COVID-19 case-fatality and mortality rates in the United States. The Innovation. 2020;1(3):100047. doi: 10.1016/j.xinn.2020.100047 32984861 PMC7505160

[59] Petroni M, Hill D, Younes L, Barkman L, Howard S, Howell IB, et al. Hazardous air pollutant exposure as a contributing factor to COVID-19 mortality in the United States. Environmental Research Letters. 2020;15(9):0940a9. doi: 10.1088/1748-9326/abaf86

[60] Karimi B, Moradzadeh R, Samadi S. Air pollution and COVID-19 mortality and hospitalization: An ecological study in Iran. Atmospheric Pollution Research. 2022; p. 101463. doi: 10.1016/j.apr.2022.101463 35664828 PMC9154086

[61] Chen Z, Huang BZ, Sidell MA, Chow T, Eckel SP, Pavlovic N, et al. Near-roadway air pollution associated with COVID-19 severity and mortality–Multiethnic cohort study in Southern California. Environment international. 2021;157:106862. doi: 10.1016/j.envint.2021.106862 34507232 PMC8416551

[62] Kogevinas M, Castaño-Vinyals G, Karachaliou M, Espinosa A, de Cid R, Garcia-Aymerich J, et al. Ambient air pollution in relation to SARS-CoV-2 infection, antibody response, and COVID-19 disease: a cohort study in Catalonia, Spain (COVICAT study). Environmental health perspectives. 2021;129(11):117003. doi: 10.1289/EHP9726 34787480 PMC8597405

[63] Tian F, Liu X, Chao Q, Qian ZM, Zhang S, Qi L, et al. Ambient air pollution and low temperature associated with case fatality of COVID-19: a nationwide retrospective cohort study in China. The Innovation. 2021;2(3):100139. doi: 10.1016/j.xinn.2021.100139 34189495 PMC8226106

[64] Elliott J, Bodinier B, Whitaker M, Delpierre C, Vermeulen R, Tzoulaki I, et al. COVID-19 mortality in the UK Biobank cohort: revisiting and evaluating risk factors. European journal of epidemiology. 2021;36(3):299–309. doi: 10.1007/s10654-021-00722-y 33587202 PMC7882869

[65] López-Feldman A, Heres D, Marquez-Padilla F. Air pollution exposure and COVID-19: A look at mortality in Mexico City using individual-level data. Science of The Total Environment. 2020;756:143929. doi: 10.1016/j.scitotenv.2020.143929 33302074 PMC7688431

[66] Pegoraro V, Heiman F, Levante A, Urbinati D, Peduto I. An Italian individual-level data study investigating on the association between air pollution exposure and Covid-19 severity in primary-care setting. BMC Public Health. 2021;21(1):1–11. doi: 10.1186/s12889-021-10949-9 33980180 PMC8114667

[67] Bozack A, Pierre S, DeFelice N, Colicino E, Jack D, Chillrud SN, et al. Long-Term Air Pollution Exposure and COVID-19 Mortality: A Patient-Level Analysis from New York City. American journal of respiratory and critical care medicine. 2021;(ja). 10.1164/rccm.202104-0845OC PMC12042910 34881681

[68] Chen C, Wang J, Kwong J, Kim J, van Donkelaar A, Martin RV, et al. Association between long-term exposure to ambient air pollution and COVID-19 severity: a prospective cohort study. CMAJ. 2022;194(20):E693–E700. doi: 10.1503/cmaj.220068 35609912 PMC9188786

[69] Sheridan C, Klompmaker J, Cummins S, James P, Fecht D, Roscoe C. Associations of air pollution with COVID-19 positivity, hospitalisations, and mortality: Observational evidence from UK Biobank. Environmental Pollution. 2022; p. 119686. doi: 10.1016/j.envpol.2022.119686 35779662 PMC9243647

[70] Nobile F, Michelozzi P, Ancona C, Cappai G, Cesaroni G, Davoli M, et al. Air pollution, SARS-CoV-2 incidence and COVID-19 mortality in Rome: a longitudinal study. European Respiratory Journal. 2022;60(3). doi: 10.1183/13993003.00589-2022 35896215 PMC9301936

[71] Veronesi G, De Matteis S, Calori G, Pepe N, Ferrario MM. Long-term exposure to air pollution and COVID-19 incidence: a prospective study of residents in the city of Varese, Northern Italy. Occupational and environmental medicine. 2022;79(3):192–199. doi: 10.1136/oemed-2021-107833 35012995

[72] Copat C, Cristaldi A, Fiore M, Grasso A, Zuccarello P, Santo Signorelli S, et al. The role of air pollution (PM and NO2) in COVID-19 spread and lethality: a systematic review. Environmental research. 2020;191:110129. doi: 10.1016/j.envres.2020.110129 32853663 PMC7444490

[73] Ali N, Islam F. The effects of air pollution on COVID-19 infection and mortality—A review on recent evidence. Frontiers in public health. 2020; p. 779. doi: 10.3389/fpubh.2020.580057 33324598 PMC7725793

[74] Srivastava A. COVID-19 and air pollution and meteorology-an intricate relationship: a review. Chemosphere. 2021;263:128297. doi: 10.1016/j.chemosphere.2020.128297 33297239 PMC7487522

[75] Bourdrel T, Annesi-Maesano I, Alahmad B, Maesano CN, Bind MA. The impact of outdoor air pollution on COVID-19: a review of evidence from in vitro, animal, and human studies. European respiratory review. 2021;30(159). doi: 10.1183/16000617.0242-2020 33568525 PMC7879496

[76] Marquès M, Domingo JL. Positive association between outdoor air pollution and the incidence and severity of COVID-19. A review of the recent scientific evidences. Environmental Research. 2022;203:111930. doi: 10.1016/j.envres.2021.111930 34425111 PMC8378989

[77] Ali N, Fariha KA, Islam F, Mishu MA, Mohanto NC, Hosen MJ, et al. Exposure to air pollution and COVID-19 severity: A review of current insights, management, and challenges. Integrated Environmental Assessment and Management. 2021;17(6):1114–1122. doi: 10.1002/ieam.4435 33913626 PMC8239695

[78] Zang ST, Luan J, Li L, Yu HX, Wu QJ, Chang Q, et al. Ambient air pollution and COVID-19 risk: evidence from 35 observational studies. Environmental research. 2022;204:112065. doi: 10.1016/j.envres.2021.112065 34534520 PMC8440008

[79] Buckee CO, Balsari S, Chan J, Crosas M, Dominici F, Gasser U, et al. Aggregated mobility data could help fight COVID-19. Science. 2020;. doi: 10.1126/science.abb8021 32205458

[80] Bernal JL, Andrews N, Gower C, Robertson C, Stowe J, Tessier E, et al. Effectiveness of the Pfizer-BioNTech and Oxford-AstraZeneca vaccines on covid-19 related symptoms, hospital admissions, and mortality in older adults in England: test negative case-control study. bmj. 2021;373. 10.1136/bmj.n1088 PMC8116636 33985964

[81] Haas EJ, McLaughlin JM, Khan F, Angulo FJ, Anis E, Lipsitch M, et al. Infections, hospitalisations, and deaths averted via a nationwide vaccination campaign using the Pfizer–BioNTech BNT162b2 mRNA COVID-19 vaccine in Israel: a retrospective surveillance study. The Lancet Infectious Diseases. 2021;. doi: 10.1016/S1473-3099(21)00566-1 34562375 PMC8457761

[82] Self WH, Tenforde MW, Rhoads JP, Gaglani M, Ginde AA, Douin DJ, et al. Comparative effectiveness of Moderna, Pfizer-BioNTech, and Janssen (Johnson & Johnson) vaccines in preventing COVID-19 hospitalizations among adults without immunocompromising conditions—United States, March–August 2021. Morbidity and Mortality Weekly Report. 2021;70(38):1337. doi: 10.15585/mmwr.mm7038e1 34555004 PMC8459899

[83] Tartof SY, Slezak JM, Fischer H, Hong V, Ackerson BK, Ranasinghe ON, et al. Effectiveness of mRNA BNT162b2 COVID-19 vaccine up to 6 months in a large integrated health system in the USA: a retrospective cohort study. The Lancet. 2021;398(10309):1407–1416. doi: 10.1016/S0140-6736(21)02183-8 PMC8489881 34619098

[84] Sun Y, Monnat SM. Rural-urban and within-rural differences in COVID-19 vaccination rates. The Journal of Rural Health. 2021;. doi: 10.1111/jrh.12625 34555222 PMC8661570

[85] Brown CC, Young SG, Pro GC. COVID-19 vaccination rates vary by community vulnerability: A county-level analysis. Vaccine. 2021;39(31):4245–4249. doi: 10.1016/j.vaccine.2021.06.038 34167838 PMC8215509

[86] Murthy BP, Sterrett N, Weller D, Zell E, Reynolds L, Toblin RL, et al. Disparities in COVID-19 vaccination coverage between urban and rural counties—United States, December 14, 2020–April 10, 2021. Morbidity and Mortality Weekly Report. 2021;70(20):759. doi: 10.15585/mmwr.mm7020e3 34014911 PMC8136424

[87] Wang S, Gao S, Li S, Feng K. Strategizing the relation between urbanization and air pollution: Empirical evidence from global countries. Journal of Cleaner Production. 2020;243:118615. doi: 10.1016/j.jclepro.2019.118615

[88] Currie J, Voorheis J, Walker R. What caused racial disparities in particulate exposure to fall? New evidence from the Clean Air Act and satellite-based measures of air quality. National Bureau of Economic Research; 2020.

[89] Makar M, Antonelli J, Di Q, Cutler D, Schwartz J, Dominici F. Estimating the causal effect of fine particulate matter levels on death and hospitalization: are levels below the safety standards harmful? Epidemiology (Cambridge, Mass). 2017;28(5):627. 28768298 10.1097/EDE.0000000000000690 PMC5624531

[90] World Health Organization. WHO Global Air Quality Guidelines: Particulate Matter (PM2.5 and PM10), Ozone, Nitrogen Dioxide, Sulfur Dioxide and Carbon Monoxide. World Health Organization. 2021;p. xxi–273. 34662007

