# Supplementary material for: Combining aggregate and individual-level data to estimate individual-level associations between air pollution and COVID-19 mortality in the United States
Source: PLOS Glob Public Health. 2023 Aug 2;3(8):e0002178. doi: 10.1371/journal.pgph.0002178 (PMC10395946; doi:10.1371/journal.pgph.0002178)
Supplement: S1 File — (PDF) [file pgph.0002178.s001.pdf]

S1 File

Combining aggregate and individual-level data to estimate individual-level associations between air pollution and COVID-19 mortality in the United States

Sophie M. Woodward, Daniel Mork, Xiao Wu, Zhewen Hou, Danielle Braun, Francesca Dominici

The Public Use Microdata Sample (PUMS) files are a set of records from individual people or housing units. The files contain a sample of the responses to the ACS that is used to create the summary and detail tables the Census publishes. Disclosure protection has been enabled to protect the confidentiality of the survey respondents. There are two types of PUMS files, one for Person records and one for Housing Unit records. Each record in the Person file represents a single person. Individuals are organized into households, making possible the study of people within the contexts of their families and other household members. The Housing Unit files contain records for single housing units, including vacant housing units. PUMS files for an individual year contain data on approximately one percent of the United States population. PUMS files covering a five-year period contain data on a nationally representative sample of approximately 5% of the United States population. For more details see [1].

The most detailed unit of geography contained in the PUMS files is the Public Use Microdata Area (PUMA). PUMAs are non-overlapping areas that partition each state into contiguous geographic units containing roughly $100,000$ people at the time of their creation. PUMAs Geographic equivalency files were provided by the US Census to show the relationship between the PUMAs and counties, and make the linkage of PUMS data with other county-level data feasible.

References

1. ACS. 2015-2019 5-YEAR Public Use Microdata Sample FILES ReadMe. US Census Bureau. 2021;.
