# Supplementary material for: Combining aggregate and individual-level data to estimate individual-level associations between air pollution and COVID-19 mortality in the United States
Source: PLOS Glob Public Health. 2023 Aug 2;3(8):e0002178. doi: 10.1371/journal.pgph.0002178 (PMC10395946; doi:10.1371/journal.pgph.0002178)
Supplement: S2 File — (PDF) [file pgph.0002178.s002.pdf]

S2 File

Combining aggregate and individual-level data to estimate individual-level associations between air pollution and COVID-19 mortality in the United States

Sophie M. Woodward, Daniel Mork, Xiao Wu, Zhewen Hou, Danielle Braun, Francesca Dominici

We specify a Gaussian prior with mean 0 and variance 0.68 for each of the regression coefficient parameters $\{\boldsymbol{\alpha}, \boldsymbol{\beta}\}$, except for the coefficients for female and age ≥ 40. Similarly, we specify a Gaussian prior with mean 0 and variance 0.68 for each of the regression coefficient parameters ω_h. These prior choices reflect a 95% prior belief that each odds ratio is between 1/5 and 5 [1, 2]. We model the baseline COVID-19 death risk for each state as $\mu_{s_i} \sim \mathcal{N}(\mu, \sigma^2)$. We specify the prior $\mu \sim \mathcal{N}(-8, 5)$ and $\sigma \sim \text{Uniform}(0, 1)$ to reflect the large variation of baseline COVID-19 death risk for different states.

Weak identifiability often arises in hierarchical modeling with ecological data where multiple covariates at different levels are related to the same outcomes [3, 4]. Unidentifiability will result in parameter estimates that are unstable and not suitable for inference without additional checks for validity through modeling reparameterizations or refined computational approaches [4]. Whereas this issue may cause unstable coefficient estimates for those weak identifiable variables, it does not necessarily invalidate the coefficient estimates for other identifiable variables. In particular, the coefficient estimate for $PM_{2.5}$, the primary interest in our study, remains identifiable and is robust to sensitivity analyses.

To overcome the weak identifiability problem in the female and age coefficients, we choose Gaussian priors for the female coefficient and age ≥ 40 coefficient based on Provisional COVID-19 Deaths by Sex and Age from the National Center for Health Statistics. We calculate the ORs for females and age ≥ 40 as 0.537 and 56.430 respectively. Indeed, these calculations are supported by [5–11] which estimate higher COVID-19 mortality and case fatality rates in men and those of older ages. These prior choices reflect a 95% prior belief that the female odds ratio is between 0.507 and 0.570, and a 95% prior belief that the age ≥ 40 odds ratio is between 54.484 and 58.445. The associated standard errors for the log ORs were 0.0302 and 0.0179. Thus, we placed the priors $\mathcal{N}(-0.621, 0.030^2)$ and $\mathcal{N}(4.033, 0.018^2)$ on the log odds ratios of female and age ≥ 40, respectively in all analyses that estimate these two coefficients.

References

1. Jackson C, Best N, Richardson S. Improving ecological inference using individual-level data. Statistics in medicine. 2006;25(12):2136–2159.

2. Jackson C, Best N, Richardson S. Hierarchical related regression for combining aggregate and individual data in studies of socio-economic disease risk factors. Journal of the Royal Statistical Society: Series A (Statistics in Society). 2008;171(1):159–178.

3. Wakefield J. Ecological inference for 2× 2 tables. Techincal Report, Department of Statistics and Biostatistics, University of Washington, USA. 2001;.

4. Ogle K, Barber JJ. Ensuring identifiability in hierarchical mixed effects Bayesian models. Ecological Applications. 2020;30(7):e02159.

5. Gomez JMD, Du-Fay-de Lavallaz JM, Fugar S, Sarau A, Simmons JA, Clark B, et al. Sex differences in COVID-19 hospitalization and mortality. Journal of Women's health. 2021;30(5):646–653.

6. Wei X, Xiao YT, Wang J, Chen R, Zhang W, Yang Y, et al. Sex differences in severity and mortality among patients with COVID-19: evidence from pooled literature analysis and insights from integrated bioinformatic analysis. arXiv preprint arXiv:200313547. 2020;.

7. Alkhouli M, Nanjundappa A, Annie F, Bates MC, Bhatt DL. Sex differences in case fatality rate of COVID-19: insights from a multinational registry. In: Mayo clinic proceedings. vol. 95. Elsevier; 2020. p. 1613–1620.

8. Parohan M, Yaghoubi S, Seraji A, Javanbakht MH, Sarraf P, Djalali M. Risk factors for mortality in patients with Coronavirus disease 2019 (COVID-19) infection: a systematic review and meta-analysis of observational studies. The Aging Male. 2020;23(5):1416–1424.

9. Mehra MR, Desai SS, Kuy S, Henry TD, Patel AN. Cardiovascular disease, drug therapy, and mortality in Covid-19. New England Journal of Medicine. 2020;382(25):e102.

10. Bonanad C, García-Blas S, Tarazona-Santabalbina F, Sanchis J, Bertomeu-González V, Facila L, et al. The effect of age on mortality in patients with COVID-19: a meta-analysis with 611,583 subjects. Journal of the American Medical Directors Association. 2020;21(7):915–918.

11. Albitar O, Ballouze R, Ooi JP, Ghadzi SMS. Risk factors for mortality among COVID-19 patients. Diabetes research and clinical practice. 2020;166:108293.
